# Association between endometrial thickness and neonatal outcomes in intrauterine insemination cycles: a retrospective analysis of 1,016 live-born singletons

**DOI:** 10.1186/s12958-020-00597-w

**Published:** 2020-05-14

**Authors:** Jialyu Huang, Jiaying Lin, Xuefeng Lu, Hongyuan Gao, Ning Song, Renfei Cai, Yanping Kuang

**Affiliations:** 1 Department of Assisted Reproduction, Shanghai Ninth People’s Hospital, Shanghai Jiao Tong University School of Medicine, 639 Zhizaoju Rd, Shanghai, 200011 China; 2 Department of Histology, Embryology, Genetics and Developmental Biology, Shanghai Key Laboratory for Reproductive Medicine, Shanghai Jiao Tong University School of Medicine, Shanghai, 200025 China

**Keywords:** Endometrial thickness, Neonatal outcomes, Gestational age, Birthweight, Intrauterine insemination

## Abstract

**Background:**

Decreased endometrial thickness (EMT) has been suggested to be associated with reduced birthweight of in vitro fertilization/intracytoplasmic sperm injection (IVF/ICSI) newborns. Considering the differences in ovarian stimulation degree and laboratory procedures between IVF/ICSI and IUI treatment, we aim to investigate whether EMT has any influence on IUI infant outcomes as well.

**Methods:**

This was a retrospective cohort study of 1016 patients who had singleton livebirths after IUI treatment cycles from January 2008 to December 2018 at a tertiary-care academic medical center in China. Patients were categorized into three groups by the 10th and 90th percentile of peak EMT: ≤7.6, 7.7–13.0 and ≥ 13.1 mm. The primary outcomes of the study were preterm birth (PTB), low birthweight (LBW) and small-for-gestational age (SGA). Multiple regression analyses were performed after controlling for a variety of potential confounders.

**Results:**

No significant differences were found among the three groups in gestational age, birthweight and birthweight Z-score. Compared with the EMT 7.7–13.0 mm group, the incidences of PTB, LBW and SGA were 5.5% (adjusted odds ratio [aOR] 0.81, 95% confidence interval [CI] 0.33–2.01), 6.4% (aOR 1.44, 95% CI 0.58–3.58) and 7.3% (aOR 1.21, 95% CI 0.53–2.76) in the EMT ≤7.6 mm group, respectively. Similarly, EMT ≥13.1 mm was not significantly associated with risks of PTB (aOR 0.63, 95% CI 0.24–1.65), LBW (aOR 0.57, 95% CI 0.17–1.95) and SGA (aOR 0.73, 95% CI 0.28–1.92). The odds of other adverse neonatal outcomes, including macrosomia, large-for-gestational age and major congenital malformations, did not show significant differences before and after adjustment in both EMT ≤7.6 and ≥ 13.1 mm groups.

**Conclusions:**

EMT is not independently associated with adverse perinatal outcomes in IUI cycles. This novel finding would provide reassuring information for IUI patients with thin endometrial linings regarding their neonatal health. However, further prospective cohort studies with larger datasets are needed to confirm the conclusion.

## Background

Intrauterine insemination (IUI) has been widely applied for infertility treatment with the reported clinical pregnancy rate varying from 5 to 20% [1–3]. However, compared with spontaneously conceived children, singletons born after IUI have shown increased risks of adverse perinatal outcomes including preterm birth (PTB), low birthweight (LBW) and small-for-gestational age (SGA) [4–7]. The major concern is whether these poorer outcomes are associated with the IUI treatment per se or intrinsic parental characteristics related to infertility [6, 8]. Understanding the relative contribution of the multifarious factors would be essential for assessing the safety of assisted reproductive treatment and promoting its continuous advancements in clinical practice.

Adequate endometrial development is of paramount importance not only for early embryo development and implantation, but also for placentation and fetal growth [9–11]. As the most commonly used indicator for endometrial receptivity, endometrial thickness (EMT) measurement has become part of standard monitoring during infertility treatment due to its simple and non-invasive approach through transvaginal ultrasonography (TVU) [12, 13]. A thin endometrium, either in fresh or frozen-thawed embryo transfer (FET) cycles, is associated with a lower chance to achieve pregnancy [12, 13]. More recently, a growing body of evidence has also suggested the possible relationship between decreased EMT and reduced birthweight of in vitro fertilization/intracytoplasmic sperm injection (IVF/ICSI) newborns [11, 14–17].

Contrary to the suboptimal endometrial environment caused by supraphysiologic hormonal milieu during IVF/ICSI cycles [18, 19], ovarian stimulation is milder in IUI treatment with fewer mature follicles and lower estradiol (E_2_) production. In addition, considering the potential effects of embryo culture medium, cryopreservation method and other laboratory procedures in IVF/ICSI [6, 8, 20, 21], a study on neonatal outcomes after IUI may be more suitable to investigate the independent influence of parental factors such as EMT. Indeed, results on EMT and pregnancy chances following IUI treatment have been controversial, with two recent meta-analyses concluding no evidence for an association [13, 22]. However, it is still unclear whether EMT has any impact on IUI infant outcomes as in IVF/ICSI cycles.

The aim of the present study was to comprehensively evaluate the association between EMT and neonatal outcomes of live-born singletons in IUI cycles.

## Methods

### Study design and population

This was a retrospective cohort study conducted at the Department of Assisted Reproduction of Shanghai Ninth People’s Hospital affiliated with Shanghai Jiao Tong University School of Medicine. The study protocol was approved by the hospital’s Ethics Committee (Institutional Review Board) and all couples provided written informed consent before the start of treatment. Infertile patients who underwent IUI cycles and had singleton livebirths after ≥24 weeks gestation were enrolled from January 2008 to December 2018. All women had at least one patent fallopian tube as determined by either hysterosalpingography or laparoscopy, and all male partners had a total motile sperm count (TMSC) of at least 10 million in the ejaculate. To minimize the likelihood of confounding [22, 23], only cycles using letrozole (LE) or in combination with human menopausal gonadotropin (hMG) for ovarian stimulation were included for analysis. We excluded patients who were previously diagnosed with congenital (i.e., uterus unicornis, septate uterus, duplex uterus and uterus bicomis) or acquired (i.e., intrauterine adhesion, endometrial polyps, submucosal myomas and adenomyosis) uterine anomalies by TVU or hysteroscopy. Cycles lost to follow-up or with core data missing in the electronic medical records (e.g., EMT) were also excluded. For patients who had more than one delivery during the study period, only the first livebirths were retained.

### Ovarian stimulation and EMT measurement

Ovarian stimulation was initiated from the 3rd day of a spontaneous or induced menstrual cycle with oral administration of LE (Jiangsu Hengrui Medicine Co., China) 2.5 mg/day for 5 days. The development of ovarian follicles was monitored by TVU measurement of mean follicular diameter and serum analysis of E_2_ and luteinizing hormone (LH) concentrations every 2 days from cycle day 10 onward. If the leading follicle size was < 14 mm on the 10th day, a daily intramuscular injection of 75 IU hMG (Anhui Fengyuan Pharmaceutical Co., China) was added for a variable duration depending on response, while no other stimulation drugs were used if the dominant follicle reached ≥14 mm. When at least one mature follicle reached a diameter of 18 mm with serum LH level < 15 IU/L, ovulation was induced by 5000 IU human chorionic gonadotropin (hCG; Lizhu Pharmaceutical Trading Co., China) and IUI was performed 36–38 h later. In cases of spontaneous LH surges (LH ≥15 IU/L), the hCG triggering was scheduled in the same afternoon followed by IUI 24 h later. We cancelled the IUI treatment cycle when more than three dominant follicles were present.

The thickness of endometrium was measured in millimeters by doctors highly trained and experienced in ultrasound monitoring via Voluson E8 (GE Healthcare, Australia) with intracavity probes. We considered the EMT as the maximal distance between the hyperechogenic interfaces of the endometrium and the myometrium about 1 cm below the uterine fundus in the sagittal plane. No specifications existed on a minimally required EMT for conducting IUI. Peak EMT on the day of triggering was recorded and used in the present study.

### Semen preparation and insemination

Semen samples were collected by masturbation after abstinence for 3–7 days and processed within 1 h after ejaculation. After liquefaction, the semen was washed with 3-layer density gradient centrifugation using Isolate (Irvine Scientific, USA). The volume of prepared semen sample for insemination was 0.3–0.5 mL and only one insemination procedure was carried out in each cycle with a soft catheter (Cook Group, USA). After IUI, bed rest was maintained for 15 min. Luteal support was routinely offered to all patients with oral dydrogesterone (Duphaston, Abbott Biologicals, USA) 10 mg twice daily from the day after IUI for 14 days.

### Neonatal follow-up

The newborn follow-up system at our center has been previously presented in detail [24, 25]. Briefly, highly trained nurses surveyed the couples by telephone during each stage of pregnancy until delivery. Basic information was gathered through standardized questionnaire including pregnancy-related complications (i.e., gestational diabetes mellitus, hypertensive disorders and thyroid diseases), gestational weeks, mode of delivery, newborn gender, birth date and locality, birthweight as well as neonatal diseases. In cases of reporting of neonatal diseases, further interviews were carried out regarding the specific diagnosis, severity, treatment and outcome. If the sick babies were born in our university hospital, the medical records at birth with detailed physical examination and routine ultrasound examination of the brain, kidney, and heart were obtained, while written proof was acquired from the pediatrician in charge for sick babies born elsewhere. In the circumstances of failed attempts to contact the couples, the local family planning service agencies were reached for data collection. Moreover, for live-born babies with congenital malformations, a special nurse was designated to review the cases thoroughly to guarantee their consistency with the case definition of the Chinese Birth Defects Monitoring Program.

### Outcome assessment

The primary outcomes of the study were PTB, LBW and SGA. Other analyzed adverse neonatal outcomes included very PTB, very LBW, macrosomia, large-for-gestational age (LGA) and major congenital malformations. PTB and very PTB were identified as delivery before 37 and 32 completed gestational weeks, respectively. LBW and very LBW were respectively defined as birthweight less than 2500 g and 1500 g, while infants with birthweight over 4000 g were categorized as macrosomia. For the standardization of birthweight, Z-score was calculated after controlling for gestational age and infant gender, based on the general population reference values of Chinese singletons [26]. In addition, SGA and LGA were defined as birthweight <10th and > 90th percentiles, respectively. Major congenital malformations were defined according to the Q codes (Q00–Q99) of the 10th Revision of International Classification of Diseases.

### Statistical analysis

All cycles were divided into three groups according to peak EMT at trigger day: ≤7.6, 7.7–13.0 and ≥ 13.1 mm. The stratification was determined using the cut-offs by the 10th (7.6 mm) and 90th (13.1 mm) percentile of the whole EMT distribution, and was also consistent with previous studies [11, 16, 27]. Continuous variables were presented as mean with standard deviation, and Kruskal-Wallis test was used for comparison since none of them showed normality after graphical illustration of histograms and Q-Q plots as well as the Shapiro-Wilk test. Categorical variables were described as frequency with rate, and between-group differences were assessed by Chi-square test or Fisher’s exact test, as appropriate.

Univariable and multivariable linear regression analyses were performed to assess the association between EMT and newborn parameters including gestational age, birthweight and Z-score, whereas logistic regression analyses were used to evaluate the effect on the incidence of adverse neonatal outcomes. The same set of potential confounders was introduced in the regression models for adjustment by the enter method, whether or not statistical differences were discovered among groups. These included maternal age (continuous), maternal body mass index (BMI) (continuous), paternal age (continuous), paternal BMI (continuous), gravidity (0 or ≥ 1), parity (0 or ≥ 1), duration of infertility (continuous), infertility diagnosis (anovulation, male factor, endometriosis, mixed or unexplained), rank of IUI attempts (1st cycle, 2nd cycle or ≥ 3rd cycle), stimulation protocol (LE or LE + hMG), length of treatment (continuous), peak E_2_ level (continuous), postprocessing TMSC (continuous), vanishing twin syndrome (VTS) (yes or no), pregnancy complications (yes or no) and year of treatment (2008–2011, 2012–2015 or 2016–2018).

All *P* values of less than 0.05 based on two-sided tests were considered to be statistically significant. Statistical analysis was performed with the Statistical Package for the Social Sciences (version 20.0; SPSS Inc., USA).

## Results

**Fig. 1:**
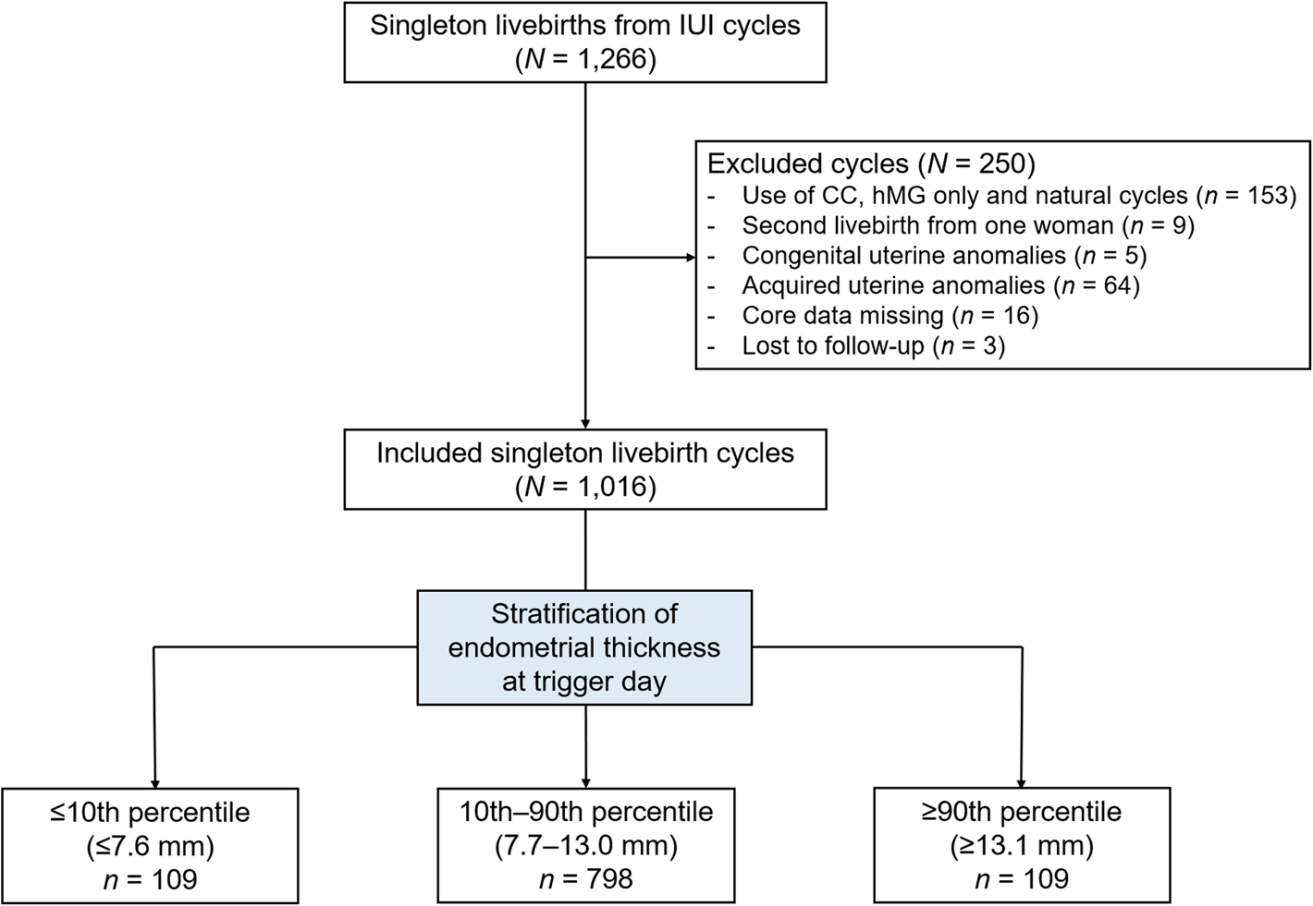
Flow chart of the study. IUI, intrauterine insemination; CC, clomiphene citrate; hMG, human menopausal gonadotropin

A total of 1266 singleton livebirths resulting from IUI were screened from our database and 250 cycles were excluded as detailed in Fig. 1. Of the remaining 1016 cycles, thin endometrium (EMT ≤7.6 mm) was observed in 109 (10.7%) women, while the number of patients with EMT 7.7–13.0 mm and ≥ 13.1 mm was 798 (78.5%) and 109 (10.7%), respectively.

Baseline characteristics according to EMT stratification were shown in Table 1. There was a significant increase from EMT ≤7.6 mm to EMT ≥13.1 mm group in the proportion of nulligravida, length of treatment and peak E_2_ level (all *P* < 0.001). No significant differences were found when parental age and BMI, parity, duration of infertility, infertility diagnosis, rank of IUI attempts, stimulation protocol, postprocessing TMSC, incidence of VTS and pregnancy complications, as well as year of treatment were compared among the three groups. With a range between 4.1 and 20.6 mm, the mean thickness of endometrium was 6.60 ± 0.96, 10.06 ± 1.43 and 14.42 ± 1.27 mm in the EMT ≤7.6, 7.7–13.0 and ≥ 13.1 mm groups, respectively (*P* < 0.001). The EMT in the LE group was slightly thinner than that in the LE + hMG group (9.68 ± 2.30 vs. 10.21 ± 2.28 mm, *P* = 0.016) (Supplementary Figure S1).

**Table 1:** Baseline characteristics according to endometrial thickness at trigger day

	≤7.6 mm (*n* = 109)	7.7–13.0 mm (*n* = 798)	≥13.1 mm (*n* = 109)	*P*-value
Maternal age (years)	30.7 ± 3.7	30.2 ± 3.7	30.0 ± 3.3	0.313
Maternal BMI (kg/m^2^)	21.76 ± 2.56	22.34 ± 3.35	22.13 ± 2.87	0.401
Paternal age (years)	32.9 ± 5.4	32.1 ± 4.4	32.1 ± 4.9	0.465
Paternal BMI (kg/m^2^)	25.09 ± 2.95	24.50 ± 3.08	24.35 ± 3.21	0.224
Nulligravida, *n* (%)	53 (48.6)	551 (69.0)	90 (82.6)	< 0.001
Nulliparity, *n* (%)	101 (92.7)	761 (95.4)	103 (94.5)	0.465
Duration of infertility (years)	2.8 ± 1.8	2.9 ± 2.0	3.0 ± 2.2	0.828
Infertility diagnosis, *n* (%)				0.610
Anovulation	19 (17.4)	143 (17.9)	16 (14.7)	
Male factor	21 (19.3)	192 (24.1)	28 (25.7)	
Endometriosis	0 (0)	18 (2.3)	3 (2.8)	
Mixed	3 (2.8)	26 (3.3)	5 (4.6)	
Unexplained	66 (60.6)	419 (52.5)	57 (52.3)	
Rank of IUI attempts, *n* (%)				0.652
1st cycle	70 (64.2)	485 (60.8)	61 (56.0)	
2nd cycle	30 (27.5)	246 (30.8)	35 (32.1)	
3rd or more	9 (8.3)	67 (8.4)	13 (11.9)	
Stimulation protocol, *n* (%)				0.661
LE	13 (11.9)	79 (9.9)	9 (8.3)	
LE + hMG	96 (88.1)	719 (90.1)	100 (91.7)	
Length of treatment (days)	10.3 ± 2.0	11.5 ± 2.9	12.5 ± 2.7	< 0.001
Peak E_2_ level (pg/mL)	256.6 ± 197.9	287.9 ± 205.8	346.3 ± 248.7	< 0.001
Endometrial thickness (mm)	6.60 ± 0.96	10.06 ± 1.43	14.42 ± 1.27	< 0.001
Postprocessing TMSC (millions)	27.8 ± 27.4	32.6 ± 37.4	33.2 ± 33.8	0.430
Vanishing twin syndrome, *n* (%)	4 (3.7)	15 (1.9)	2 (1.8)	0.469
Pregnancy complications, *n* (%)	7 (6.4)	60 (7.5)	9 (8.3)	0.875
Year of treatment				0.131
2008–2011	6 (5.5)	72 (9.0)	14 (12.8)	
2012–2015	39 (35.8)	345 (43.2)	43 (39.4)	
2016–2018	64 (58.7)	381 (47.7)	52 (47.7)	

Data are presented as mean ± standard deviation or number (percentage).

*BMI* body mass index, *IUI* intrauterine insemination, *LE* letrozole, *hMG* human menopausal gonadotropin, *E*_*2*_ estradiol, *TMSC* total motile sperm count

As presented in Table 2, comparisons among the three groups did not reveal any significant difference in gestational age, birthweight and birthweight Z-score (*P* = 0.503, 0.732 and 0.914, respectively). These findings were not altered in multivariable analyses after controlling for a variety of confounding factors (Table 3). Compared with the EMT 7.7–13.0 mm group, the incidences of PTB, LBW and SGA were 5.5% (adjusted odds ratio [aOR] 0.81, 95% confidence interval [CI] 0.33–2.01), 6.4% (aOR 1.44, 95% CI 0.58–3.58) and 7.3% (aOR 1.21, 95% CI 0.53–2.76) in the EMT ≤7.6 mm group, respectively. Similarly, EMT ≥13.1 mm was not significantly associated with risks of PTB (aOR 0.63, 95% CI 0.24–1.65), LBW (aOR 0.57, 95% CI 0.17–1.95) and SGA (aOR 0.73, 95% CI 0.28–1.92). With regard to other adverse neonatal outcomes including macrosomia, LGA and major congenital malformations, no significant differences were found in both EMT ≤7.6 and ≥ 13.1 mm groups before and after adjustment.

**Table 2:** Neonatal outcomes according to endometrial thickness at trigger day

	≤7.6 mm (*n* = 109)	7.7–13.0 mm (*n* = 798)	≥13.1 mm (*n* = 109)	*P*-value
Mode of delivery, *n* (%)				0.533
Vaginal	48 (44.0)	326 (40.9)	50 (45.9)	
Cesarean section	61 (56.0)	472 (59.1)	59 (54.1)	
Gender, *n* (%)				0.493
Female	60 (55.0)	395 (49.5)	52 (47.7)	
Male	49 (45.0)	403 (50.5)	57 (52.3)	
Gestational age (days)	274.3 ± 8.4	273.3 ± 11.7	274.5 ± 9.4	0.503
Preterm birth, *n* (%)	6 (5.5)	59 (7.4)	5 (4.6)	0.462
Very preterm birth, *n* (%)	0 (0)	8 (1.0)	0 (0)	0.530
Birthweight (g)	3319.8 ± 499.9	3304.3 ± 511.6	3323.3 ± 442.5	0.732
Low birthweight, *n* (%)	7 (6.4)	37 (4.6)	3 (2.8)	0.435
Very low birthweight, *n* (%)	0 (0)	6 (0.8)	0 (0)	1.000
Macrosomia, *n* (%)	6 (5.5)	54 (6.8)	4 (3.7)	0.430
Z-score	0.25 ± 1.07	0.25 ± 1.08	0.23 ± 1.01	0.914
Small-for-gestational age, *n* (%)	8 (7.3)	49 (6.1)	5 (4.6)	0.694
Large-for-gestational age, *n* (%)	19 (17.4)	123 (15.4)	13 (11.9)	0.509
Major congenital malformations, *n* (%)	4 (3.7)	10 (1.3)	2 (1.8)	0.119

Data are presented as mean ± standard deviation or number (percentage)

**Table 3:** Crude and adjusted analysis of neonatal outcomes in endometrial thickness categories

	≤7.6 vs. 7.7–13.0 mm		≥13.1 vs. 7.7–13.0 mm	
	Crude	Adjusted ^a^	Crude	Adjusted ^a^
Newborn parameters, MD (95% CI)				
Gestational age (days)	1.0 (−1.2–3.2)	0.7 (−1.5–3.0)	1.2 (−1.0–3.4)	1.5 (−0.7–3.7)
Birthweight (g)	15.6 (−85.3–116.5)	16.4 (−86.6–119.4)	19.0 (−81.9–119.9)	28.6 (−73.2–130.3)
Z-score	−0.01 (− 0.22–0.21)	0 (− 0.21–0.22)	−0.02 (− 0.23–0.20)	0 (− 0.22–0.21)
Adverse outcomes, OR (95% CI)				
Preterm birth	0.73 (0.31–1.73)	0.81 (0.33–2.01)	0.60 (0.24–1.54)	0.63 (0.24–1.65)
Low birthweight	1.41 (0.61–3.25)	1.44 (0.58–3.58)	0.58 (0.18–1.92)	0.57 (0.17–1.95)
Macrosomia	0.80 (0.34–1.91)	0.91 (0.37–2.25)	0.53 (0.19–1.48)	0.53 (0.18–1.54)
Small-for-gestational age	1.21 (0.56–2.63)	1.21 (0.53–2.76)	0.74 (0.29–1.89)	0.73 (0.28–1.92)
Large-for-gestational age	1.16 (0.68–1.97)	1.21 (0.69–2.11)	0.74 (0.40–1.37)	0.70 (0.37–1.34)
Major congenital malformations	3.00 (0.93–9.74)	3.18 (0.82–12.28)	1.47 (0.32–6.81)	1.32 (0.24–7.13)

*MD* mean difference, *CI* confidence interval, *OR* odds ratio

^a^ Adjusted for parental age, parental body mass index, gravidity, parity, infertility duration, infertility diagnosis, rank of cycle, stimulation protocol, length of treatment, peak estradiol level, postprocessing total motile sperm count, vanishing twin syndrome, pregnancy complications, and year of treatment

Multiple regression analyses of risk factors for PTB, LBW and SGA were detailed in Supplementary Table S1. Pregnancy complications were found to be the common influencing factor, while higher risks of PTB and LBW were observed in women with VTS. Duration of infertility was demonstrated to be associated with an increased risk of SGA (aOR 1.17, 95% CI 1.03–1.32).

## Discussion

In this retrospective analysis of 1016 live-born singletons, we provided the first evidence that EMT was not associated with adverse perinatal outcomes in IUI cycles. Neither a thin or thickened endometrial lining (EMT ≤7.6 or ≥ 13.1 mm) at trigger day would increase the infant risks of PTB, LBW, SGA and major congenital anomalies.

Despite significant progress in ultrasonographic, immunological and molecular diagnostic techniques of endometrial receptivity, the EMT still remains to be the mainly used marker as part of standard cycle monitoring in assisted reproductive treatment [12, 13]. Over the past decades, much efforts have been made to explore its prognostic capacity in pregnancy chances. While it has been well-established that the probability of clinical pregnancy was lower in IVF/ICSI patients with thin EMT defined by different cut-off values from 6 to 10 mm [12], only few studies have investigated this issue in the setting of IUI treatment with conflicting results [13, 22]. The pooled data revealed no significant difference in mean EMT between pregnant and non-pregnant IUI women, and no difference in clinical pregnancy was observed after association analysis using EMT cut-offs ranging from 3 to 12 mm [13, 22]. Although the discrepancy remains unclarified, it does highlight the necessity to separately assess the influence of EMT on reproductive outcomes between IUI and IVF/ICSI treatment.

The relationship between EMT and neonatal morbidity in IVF/ICSI cycles has been evaluated in several prior studies [11, 14–17]. In 2006, Chung et al. [14] first reported that patients with an EMT ≤10 mm had a two-fold higher risk of experiencing PTB, LBW or intrauterine fatal demise beyond first trimester than those with an EMT > 12 mm. This study, however, was flawed by a high proportion of multiple gestations (37%) out of 435 viable pregnancies. By retrospectively analyzing 764 singleton deliveries, a later study by Moffat et al. [15] showed that EMT was strongly predictive for neonatal birthweight in pregnancies with obstetric complications but not in uneventful pregnancies. Consistently, Ribeiro and colleagues [16] found that an EMT less than 7 mm was significantly associated with a decrease in neonatal birthweight Z-score. However, they did not detect an increase in clinically relevant outcomes such as PTB or LBW, and attributed it to the limited size of the live-born singleton sample subset. This was subsequently validated in another study by Oron et al. [11] showing that women with an EMT < 7.5 mm had a higher incidence of placenta-related pregnancy complications including SGA. In addition to the aforementioned four studies on fresh embryo transfer cycles, a recent analysis of 6181 singletons resulting from FET also demonstrated that a thin endometrium (EMT < 8 mm) was independently related to a lower birthweight and Z-score, while the modest difference failed to translate into a significantly higher risk of SGA [17].

Similar to the contradictory findings on pregnancy outcomes, the present study reported no evidence for an association between EMT and IUI infant outcomes as in IVF/ICSI cycles. However, the underlying reasons are still unclear as to why a thin endometrial lining is more clinically relevant in IVF/ICSI than in IUI treatment. During either natural or stimulated cycles, continuous E_2_ production from growing follicles induces endometrial proliferation, thus leading to increased EMT as measured by TVU. Compared with a thin endometrium developing under mild ovarian stimulation conditions as in IUI, a thin endometrium developing under maximal stimulation conditions in IVF/ICSI cycles may imply genuine diminished potential for implantation and placentation. Moreover, in the presence of thin endometrium, the embryos are implanted much closer to the spiral arteries of the endometrial basal layer, whose higher vascularity and oxygen concentrations could be detrimental to embryos due to the production of reactive oxygen species [28, 29]. Since embryos are developed in vivo in IUI cycles, we speculate that they may be less susceptible to high oxygen tensions in comparison with in vitro cultured or further vitrified/thawed embryos in IVF laboratories. Nevertheless, these explanations are merely theoretical and further investigations are warranted to provide stronger and more direct evidence.

VTS, defined as the spontaneous reduction of multiple pregnancies to singleton birth [30], was identified as an independent predictor of adverse neonatal outcomes after multivariable regression analysis. This phenomenon has been estimated to occur in 50% of pregnancies with initially three or more gestational sacs, 36% of twin pregnancies and 10–30% of IVF/ICSI pregnancies [31, 32]. In the present study, we reported for the first time that the prevalence of VTS was about 2.1% in IUI treatment, much lower than that in IVF/ICSI cycles. Consistent with previous findings on IVF/ICSI infants [30, 33, 34], VTS is associated with higher risks of PTB and LBW in IUI newborns as well. However, the adjusted odds of SGA and birth defects did not reach statistical significance possibly due to the limitation of relatively small sample size, as reflected by the wide confidence intervals. Therefore, more studies with larger datasets are needed to further assess the incidence, risk factors as well as neonatal outcomes of VTS in IUI cycles.

A major weakness of the study relies on its retrospective design. Some baseline characteristics were not balanced between the stratified groups. Notably, the fact that more patients with thin EMT had been pregnant before but the frequency of nulliparity was similar among the three groups suggested a higher rate of prior pregnancy loss, which was associated with increased chances of adverse neonatal outcomes such as PTB and LBW [35]. While the present study included gravidity and parity number for adjustment, we were unable to retrieve data on the actual causes of pregnancy loss history (e.g., miscarriage, abortion or ectopic pregnancy). In addition, possible unknown or unavailable confounders may not be accounted for in the regression model. For example, over 50% of singletons were delivered via cesarean section, but the indications and timing in relation to neonatal mortality remained unclear [36, 37]. Also, information on endometrial morphology, which has been reported to influence pregnancy outcomes [13], was incomplete in our database and thus not analyzed in this study. Other risk factors for adverse neonatal outcomes, including previous delivery history, medication use, metabolic status, nutrition intake and lifestyle habit [38], were not adequately controlled due to the lack of detailed records. Therefore, future studies with a comprehensive evaluation of women before and during pregnancy are needed for further investigation. Secondly, previous studies have observed significant differences in EMT among various ovarian stimulation protocols and in neonatal outcomes of different sperm sources [7, 22, 23, 39]. To minimize the likelihood of confounding, only IUI cycles using LE and partner semen were included for analysis. Thus, our conclusion should not be extrapolated to IUI singletons born after treatment with donor semen or with clomiphene citrate, gonadotropin only and natural cycles. The single-center nature should also caution generalization of the study finding to other areas in China and other parts of the world. Finally, while the measurement of EMT was performed by highly experienced doctors at our center, some inter-observer inconsistency may still be present. Moreover, most of the neonatal data were collected from parental questionnaires instead of direct access to medical records. In this regard, the detection of some minor problems could be comprised and the actual incidence of congenital malformations may be underestimated.

## Conclusions

In summary, our data suggested no association between EMT and adverse neonatal outcomes in IUI cycles. This novel finding would provide reassuring information for patients undergoing IUI treatment with thin endometrial linings regarding their neonatal health. However, further large prospective cohort studies with longer follow-up duration are needed to confirm the conclusion.

## Supplementary information

**Additional file 1: Supplementary Figure S1.** Distribution of peak endometrial thickness in different ovarian stimulation protocols. LE, letrozole; hMG, human menopausal gonadotropin.

**Additional file 2: Supplementary Table S1.** Multiple regression analysis of risk factors for PTB, LBW and SGA.

## Data Availability

The datasets used and/or analyzed during the current study are available from the corresponding author on reasonable request.

## Abbreviations

aOR: Adjusted odds ratio
BMI: Body mass index
CI: Confidence interval
E_2_: Estradiol
EMT: Endometrial thickness
FET: Frozen-thawed embryo transfer
hCG: Human chorionic gonadotrophin
hMG: Human menopausal gonadotropin
ICSI: Intracytoplasmic sperm injection
IUI: Intrauterine insemination
IVF: In vitro fertilization
LBW: Low birthweight
LE: Letrozole
LGA: Large-for-gestational age
LH: Luteinizing hormone
TB: Preterm birth
SGA: Small-for-gestational age
TMSC: Total motile sperm count
TVU: Transvaginal ultrasonography
VTS: Vanishing twin syndrome
