# Supplementary figures and images for: Association between endometrial thickness and neonatal outcomes in intrauterine insemination cycles: a retrospective analysis of 1,016 live-born singletons
Source: Reprod Biol Endocrinol. 2020 May 14;18:48. doi: 10.1186/s12958-020-00597-w (PMC7222451; doi:10.1186/s12958-020-00597-w)

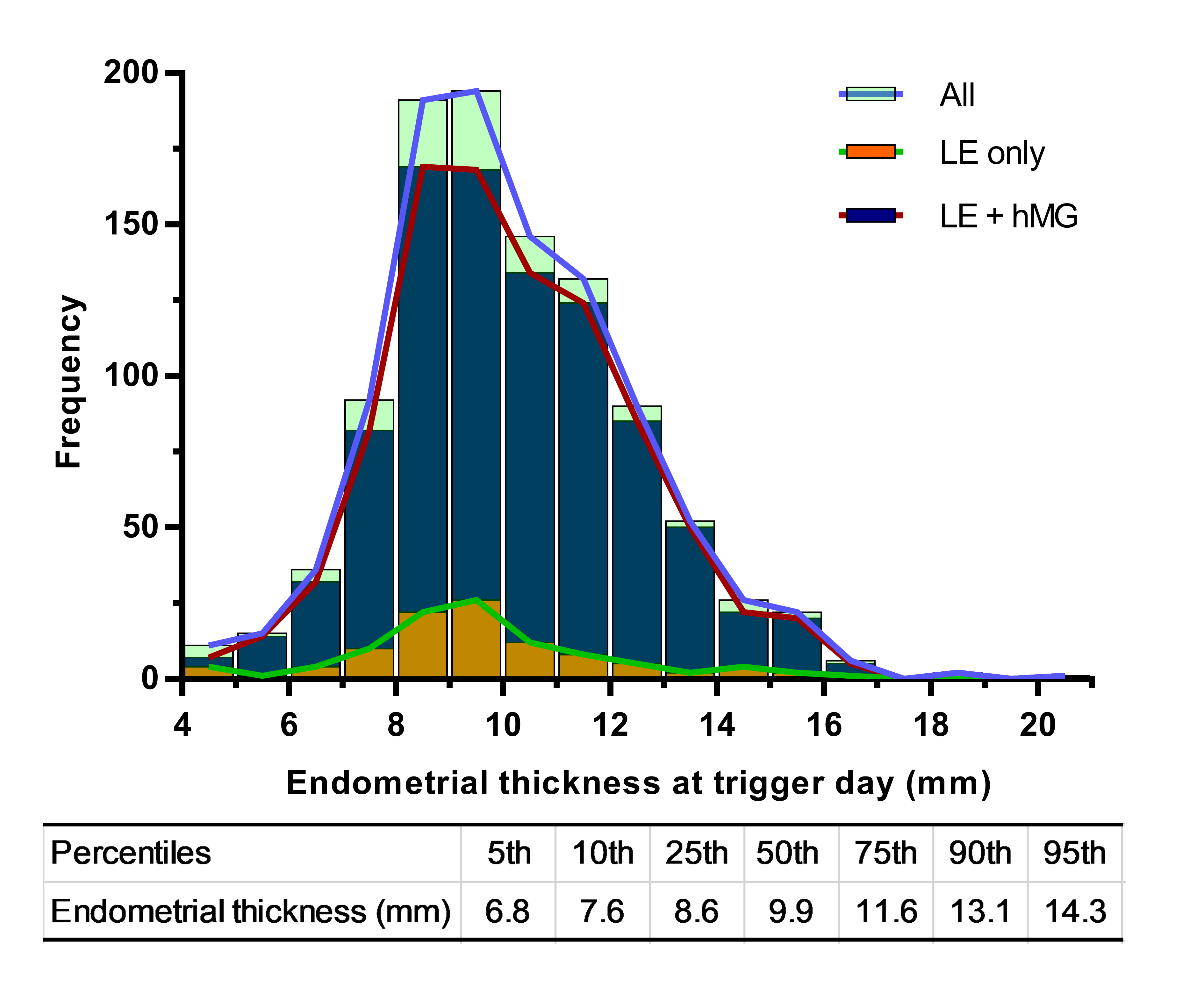

Supplement: Supplementary file 1 — Additional file 1: Supplementary Figure S1. Distribution of peak endometrial thickness in different ovarian stimulation protocols. LE, letrozole; hMG, human menopausal gonadotropin. [file 12958_2020_597_MOESM1_ESM.tif]
