# Supplementary material for: Association between endometrial thickness and neonatal outcomes in intrauterine insemination cycles: a retrospective analysis of 1,016 live-born singletons
Source: Reprod Biol Endocrinol. 2020 May 14;18:48. doi: 10.1186/s12958-020-00597-w (PMC7222451; doi:10.1186/s12958-020-00597-w)
Supplement: Supplementary file 2 — Additional file 2: Supplementary Table S1. Multiple regression analysis of risk factors for PTB, LBW and SGA. [file 12958_2020_597_MOESM2_ESM.docx]

**Supplementary Table S1.** Multiple regression analysis of risk factors for PTB, LBW and SGA.

|  | PTB | |  | LBW | |  | SGA | |
| --- | --- | --- | --- | --- | --- | --- | --- | --- |
|  | Adjusted OR  (95% CI) | *P*-value |  | Adjusted OR  (95% CI) | *P*-value |  | Adjusted OR  (95% CI) | *P*-value |
| Maternal age (per year increase) | 1.07 (0.96–1.20) | 0.225 |  | 1.02 (0.90–1.16) | 0.800 |  | 0.99 (0.88–1.11) | 0.799 |
| Maternal BMI (per kg/m^2^ increase) | 1.04 (0.95–1.13) | 0.388 |  | 1.07 (0.97–1.18) | 0.197 |  | 0.99 (0.90–1.09) | 0.870 |
| Paternal age (per year increase) | 0.97 (0.89–1.06) | 0.490 |  | 1.03 (0.94–1.14) | 0.546 |  | 0.98 (0.90–1.07) | 0.670 |
| Paternal BMI (per kg/m^2^ increase) | 0.93 (0.85–1.01) | 0.089 |  | 0.95 (0.86–1.06) | 0.385 |  | 0.97 (0.89–1.06) | 0.520 |
| Gravidity (≥1 vs. 0) | 1.12 (0.63–1.99) | 0.712 |  | 1.20 (0.60–2.39) | 0.611 |  | 1.24 (0.69–2.23) | 0.467 |
| Parity (≥1 vs. 0) | 0.73 (0.20–2.67) | 0.630 |  | 0.99 (0.26–3.75) | 0.985 |  | 0.24 (0.03–1.85) | 0.170 |
| Duration of infertility (per year increase) | 0.97 (0.89–1.06) | 0.466 |  | 1.08 (0.94–1.24) | 0.261 |  | **1.17 (1.03–1.32)** | **0.013** |
| Infertility diagnosis |  | 0.545 |  |  | 0.175 |  |  | 0.508 |
| Anovulation | Reference |  |  | Reference |  |  | Reference |  |
| Male factor | 1.24 (0.51–3.00) |  |  | 1.30 (0.37–4.52) |  |  | 0.90 (0.34–2.41) |  |
| Endometriosis | 3.27 (0.84–12.71) |  |  | 4.98 (0.81–30.82) |  |  | 2.14 (0.40–11.42) |  |
| Mixed | 1.09 (0.22–5.40) |  |  | 1.13 (0.12–10.42) |  |  | 1.37 (0.27–6.89) |  |
| Unexplained | 1.19 (0.56–2.50) |  |  | 2.62 (0.94–7.27) |  |  | 1.60 (0.73–3.51) |  |
| Rank of IUI attempts |  | 0.397 |  |  | 0.982 |  |  | 0.377 |
| 1st cycle | Reference |  |  | Reference |  |  | Reference |  |
| 2nd cycle | 0.94 (0.52–1.70) |  |  | 1.06 (0.54–2.10) |  |  | 0.82 (0.45–1.50) |  |
| 3rd or more | 1.66 (0.76–3.64) |  |  | 1.06 (0.35–3.20) |  |  | 0.44 (0.13–1.49) |  |
| Stimulation protocol (LE + hMG vs. LE) | 1.27 (0.52–3.12) | 0.607 |  | 0.85 (0.31–2.30) | 0.745 |  | 1.05 (0.42–2.62) | 0.917 |
| Length of treatment (per day increase) | 1.00 (0.91–1.11) | 0.934 |  | 1.06 (0.95–1.17) | 0.306 |  | 0.98 (0.89–1.09) | 0.763 |
| Peak E_2_ level (per log pg/mL increase) | 0.56 (0.19–1.64) | 0.292 |  | 0.55 (0.15–2.00) | 0.364 |  | 1.60 (0.53–4.86) | 0.407 |
| Endometrial thickness (mm) |  | 0.594 |  |  | 0.466 |  |  | 0.723 |
| ≤7.6 | 0.81 (0.33–2.01) |  |  | 1.44 (0.58–3.58) |  |  | 1.21 (0.53–2.76) |  |
| 7.7–13.0 | Reference |  |  | Reference |  |  | Reference |  |
| ≥13.1 | 0.63 (0.24–1.65) |  |  | 0.57 (0.17–1.95) |  |  | 0.73 (0.28–1.92) |  |
| Postprocessing TMSC  (per log million increase) | 1.50 (0.84–2.69) | 0.174 |  | 0.91 (0.46–1.83) | 0.796 |  | 0.92 (0.51–1.66) | 0.777 |
| Vanishing twin syndrome (yes vs. no) | **5.48 (1.84–16.33)** | **0.002** |  | **6.58 (1.97–21.93)** | **0.002** |  | 1.83 (0.40–8.34) | 0.434 |
| Pregnancy complications (yes vs. no) | **3.05 (1.32–7.05)** | **0.009** |  | **2.65 (1.01–6.96)** | **0.048** |  | **2.59 (1.11–6.02)** | **0.027** |
| Year of treatment |  | 0.116 |  |  | 0.326 |  |  | 0.978 |
| 2008–2011 | Reference |  |  | Reference |  |  | Reference |  |
| 2012–2015 | 0.74 (0.33–1.65) |  |  | 0.55 (0.21–1.40) |  |  | 0.95 (0.37–2.47) |  |
| 2016–2018 | 0.44 (0.18–1.05) |  |  | 0.48 (0.18–1.28) |  |  | 1.01 (0.38–2.67) |  |

PTB, preterm birth; LBW, low birthweight; SGA, small-for-gestational age; OR, odds ratio; CI, confidence interval; BMI, body mass index; IUI, intrauterine insemination; LE, letrozole; hMG, human menopausal gonadotropin; E_2_, estradiol; TMSC, total motile sperm count.
